# Obscure gastrointestinal bleeding localization using preoperative super-selective mesenteric angiography and intraoperative methylene blue injection: A case report and literature review

**DOI:** 10.1016/j.ijscr.2019.10.059

**Published:** 2019-11-01

**Authors:** Sidra B. Bhuller, Mark Lieser, Naveed Ismail, Bradley Woods

**Affiliations:** a Department of Surgery, Sky Ridge Medical Center, 10101 RidgeGate Parkway, Lone Tree, CO 80124, USA; b Department of Trauma Surgery, Research Medical Center, 2316 E Meyer Blvd, Kansas City, MO 64132, USA

**Keywords:** AVM, arteriovenous malformation, BRBPR, bright red blood per rectum, CT, computed tomography, CTA, computed tomography angiography, ED, emergency department, EGD, esophagogastroduodenoscopy, GI, gastrointestinal, Hb, hemoglobin, HD, hospital day, LTAC, long-term acute care, OGIB, obscure gastrointestinal bleeding, pRBC, packed red blood cells, SSMA, superselective mesenteric angiography, Obscure gastrointestinal bleeding, Superselective angiography, Methylene blue, Arteriovenous malformation, Angiodysplasia

## Abstract

**Introduction:**

Currently available diagnostic tests for localizing the source of gastrointestinal bleeding include esophagogastroduodenoscopy, colonoscopy, push enteroscopy, video capsule endoscopy, deep enteroscopy, nuclear scan, angiography, radiographic contrast studies of the small bowel, intraoperative enteroscopy, and computed tomography scanning. Despite these diagnostic modalities obscure gastrointestinal bleeding (OGIB) can be difficult to localize, making surgical intervention challenging.

**Presentation of case:**

We report the case of a 78-year-old patient who presented with OGIB, melena and passing bright red blood per rectum, with a hemoglobin of 4.8 g/dl requiring multiple blood transfusions. Initially the source of the bleeding was difficult to identify. Eventually, a preoperative superior mesenteric angiogram was performed, identifying an arteriovenous malformation (AVM) within the small bowel as the source of OGIB. Intraoperative methylene blue was then injected through the previously placed angiogram catheter to further localize the bowel segment with the lesion.

**Discussion:**

Despite multiple diagnostic modalities, OGIB, especially originating in the small bowel, is challenging to localize, making surgical intervention difficult. Methylene blue is frequently used intraoperatively to properly identify anatomic landmarks.

**Conclusion:**

In cases of OGIB, superselective mesenteric angiography (SSMA) with intraoperative methylene blue injection can be used as an adjunct to routine diagnostic modalities to guide surgical interventions for controlling hemorrhage and for limiting the extent of bowel resection.

## Highlights

• OGIB can be a diagnostic challenge.
• Superselective mesenteric angiography with intraoperative methylene blue injection can be used as an adjunct to pre-existing diagnostic modalities.
• Superselective mesenteric angiography can guide surgical intervention to control hemorrhage and limit the amount of bowel resected.

## 1 Introduction

Obscure gastrointestinal bleeding (OGIB) is defined as occult or overt bleeding of unknown origin that persists or recurs after an initial negative endoscopic evaluation, including colonoscopy and esophagogastroduodenoscopy (EGD) [1]. Currently available diagnostic tests for OGIB include EGD, colonoscopy, push enteroscopy, video capsule endoscopy, deep enteroscopy, nuclear scan, angiography, radiographic contrast studies of the small bowel, intraoperative enteroscopy, and computed tomography (CT) scanning; however, OGIB can still be difficult to localize, making surgical intervention challenging [[2], [3], [4]]. Potential causes of OGIB can be secondary to mass lesions (e.g., carcinoma or polyps) or inflammatory (e.g., ulcers or inflammatory bowel disease), vascular (e.g., ectasias or hemangiomas), or infectious diseases (e.g., tuberculous enteritis) [1].

**Fig. 1:**
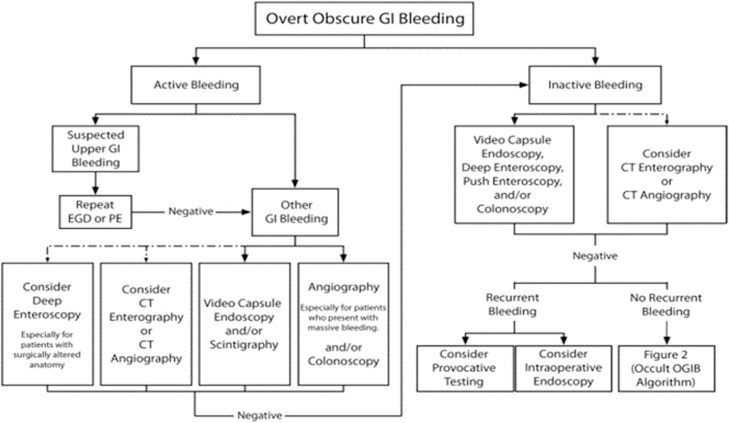
The American Society for Gastrointestinal Endoscopy’s suggested diagnostic approach to overt obscure gastrointestinal bleeding [1]. Dashed arrows indicate less-preferred options.

GI bleeding between the ligament of Treitz and the ileocecal valve accounts for approximately 5% of all GI bleeding; however, the small bowel is responsible for 45%–75% of all OGIB cases [5]. A suggested diagnostic approach to OGIB proposed by the American Society for Gastrointestinal Endoscopy is shown below in Fig. 1 [1]. Specific therapy is dictated by positive test results. Because of the synergistic nature of these diagnostic modalities, more than one test may be needed. The work has been reported in line with the SCARE criteria [6].

## 2 Presentation of case

A 78-year-old patient presented to the emergency department (ED) in hemorrhagic shock, with OGIB consisting of melena and bright red blood per rectum (BRBPR), requiring multiple transfusions and vasopressor support. The small bowel was identified as the origin of OGIB. The patient underwent preoperative localization of the small bowel bleeding site using superselective mesenteric angiography (SSMA) and intraoperative methylene blue injection, leading to a resection of approximately 10 cm of small bowel and effective hemorrhage control.

Two weeks prior to presentation, the patient was discharged from the hospital to long-term acute care (LTAC); she was previously admitted to the hospital because of traumatic injuries from an auto accident. Her injuries at the time included C2 spine fracture, C6–T2 transverse process fractures, L3 endplate fracture, right pneumothorax, bilateral ribs 1–12 fractures, grade III liver laceration, grade II splenic laceration, and right adrenal laceration. The patient underwent numerous surgical procedures, including multiple exploratory laparotomies, splenectomy, rib fixation and plating, tracheostomy, and gastrojejunostomy feeding tube placement. The patient was discharged to LTAC in a stable condition on a ventilator on hospital day (HD) 22. The patient returned to the emergency department 13 days post discharge with the above mentioned OGIB and hemoglobin (Hb) of 4.8 g/dl. The patient was transfused with 7 U of packed red blood cells (pRBC) at the LTAC prior to arrival to the ED. The patient was admitted to the intensive care unit for hemorrhagic shock; the GI bleed is thought to be unrelated to the traumatic event.

**Fig. 2:**
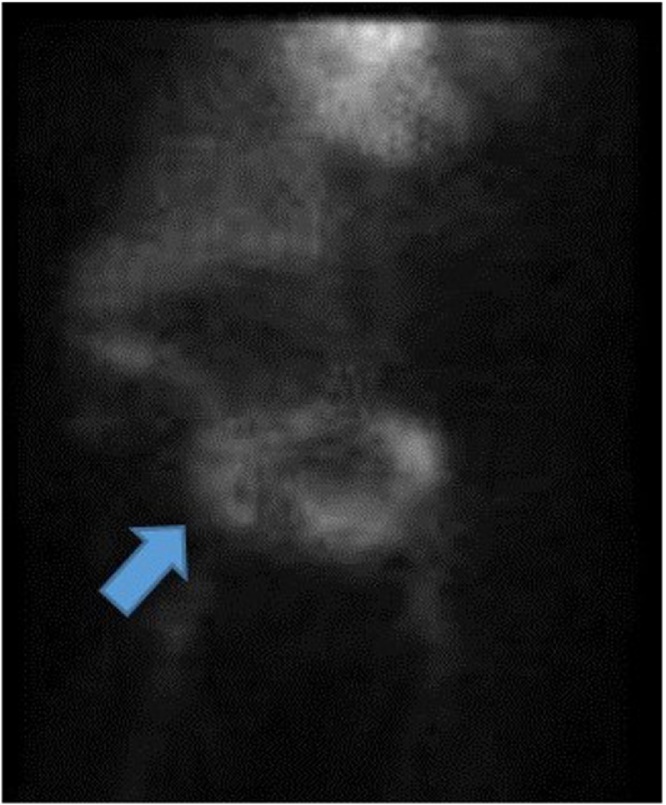
Nuclear medicine OGIB scan demonstrating active bleeding within the small bowel (blue arrow). OGIB, obscure gastrointestinal bleeding.

**Fig. 3:**
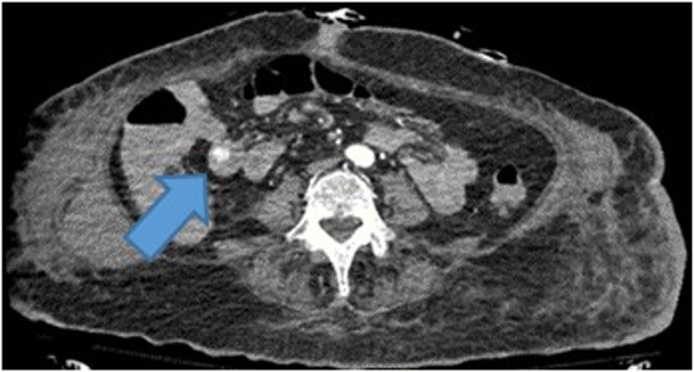
CTA of the abdomen and pelvis demonstrating active bleeding with extravasation of contrast seen within a small bowel loop in the right mid-abdomen, well proximal to the terminal ileum (blue arrow). CTA, computed tomography angiography.

**Fig. 4:**
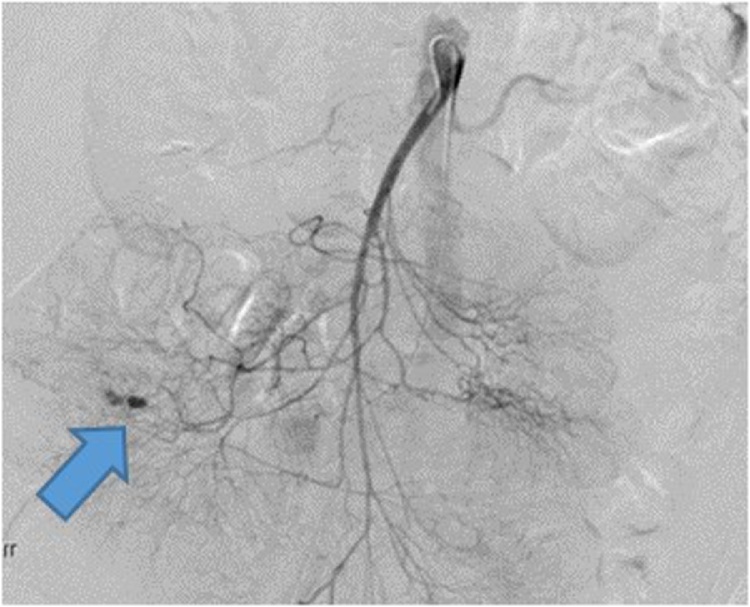
Angiography demonstrating an irregular area of contrast extravasation from a jejunal branch in the right abdomen, just below the hepatic flexure (blue arrow).

**Fig. 5:**
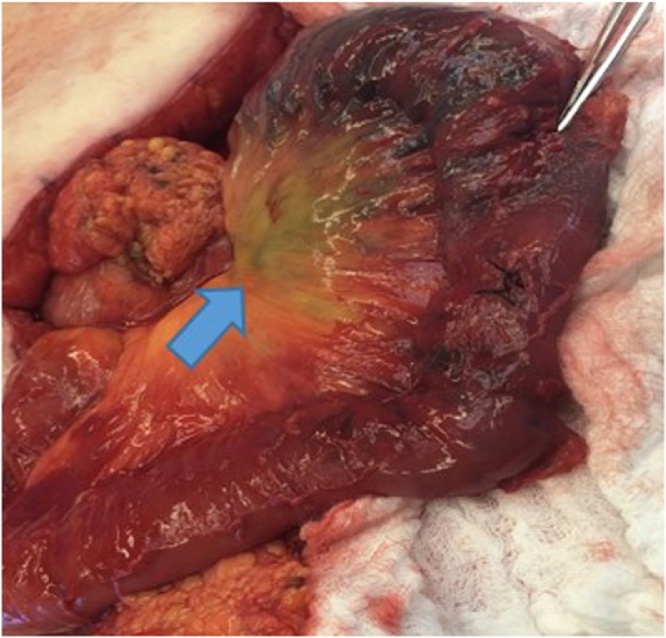
Intraoperative methylene blue injection showing area of concern and the small bowel segment subsequently resected (blue arrow).

On HD 1, a computed tomography angiography (CTA) of the abdomen and pelvis was performed without identifying an obvious source of the GIB. The patient also underwent an EGD on HD 1, which revealed diffuse duodenitis but no obvious source of active bleeding. The patient then underwent a colonoscopy and a nuclear GIB scan on HD 2, and an obvious source of bleeding was still not identified. The patient continued to have melena and BRBPR, requiring multiple transfusions. Two sequential repeat CTAs of the abdomen and pelvis and a repeat colonoscopy were obtained on HD 3, with an obvious source of GIB still not identified. Overall, the patient required 1 U pRBC on HD 1, 2 U on HD 2, 3 U on HD 3, and 9 U on HD 4. A nuclear medicine GIB scan was repeated on HD 4 because of continually dropping Hb despite multiple transfusions; this scan demonstrated active GIB within the small bowel (Fig. 2). The nuclear medicine scan was followed up with a CTA of the abdomen and pelvis, which also showed active bleeding with extravasation of contrast seen within a small bowel loop in the right mid-abdomen, well proximal to the terminal ileum (Fig. 3). A superior mesenteric angiogram was then performed in the standard fashion via the femoral artery route and identified an irregular area of contrast extravasation from a jejunal branch in the right abdomen, just below the hepatic flexure, concerning for a pseudoaneurysm or vascular malformation (Fig. 4). The microcatheter was left in place adjacent to the area of contrast extravasation to use for localization of the affected small bowel intraoperatively. The patient was then taken to the operating suite directly from interventional radiology. Intraoperatively, the previously mentioned microcatheter was used to inject 0.5 mL of methylene blue (10 mg/mL); an area of concern in a segment of small bowel mesentery immediately changed color (Fig. 5). This segment was resected, and a stapled anastomosis was performed. Postoperatively, the patient’s Hb and blood pressure stabilized, and patient did not require any additional blood products. The final pathology report indicated congested and ecstatic vasculature with thick walled submucosal vessels and mucosal surface ulceration consistent with angiodysplasia. Unfortunately, the patient developed septic shock secondary to multidrug resistant *acinetobacter baumannii* pneumonia, followed by multiorgan failure. The family withdrew care on postoperative day 2, and the patient subsequently passed away.

## 3 Discussion

Multiple diagnostic imaging modalities are available to investigate the GI tract in patients with occult bleeding. Despite this, localizing the exact source of OGIB can be challenging, particularly lesions in the small bowel. Here we used a lesser known diagnostic and therapeutic localizing method previously described by by Pai et al. [7] and Gifford et al. [8] SSMA helps narrow down the exact source of the OGIB, allowing surgeons to resect diseased bowel only. SSMA with intraoperative injection of methylene blue made identification and subsequent resection of the affected small bowel a fairly simple and quick process, compared with visually trying to identify the source of bleeding. Methylene blue, also known as methylthioninium chloride, is a medication that is frequently used intraoperatively as a dye to turn urine, sweat, lymphatics, or stool blue to green in color to properly identify anatomic landmarks. It is most commonly used in breast surgery to identify sentinel lymph nodes to detect metastatic disease and in colorectal surgery to identify the ureter to prevent injury. Headache, vomiting, confusion, shortness of breath, and high blood pressure are the most common side effects. Using preoperative SSMA with intraoperative methylene blue injection, we could resolve our patient’s bleeding, and the patient did not require any further transfusions postoperatively.

## 4 Conclusion

In cases of OGIB, SSMA with intraoperative methylene blue injection can be used as an adjunct to the pre-existing diagnostic tests to guide surgical interventions for controlling hemorrhage and limiting the extent of bowel resected.

## Funding

None.

## Ethical approval

Since this is a case report, it is exempt from ethical approval in my institution.

## Consent

Consent is available upon request.

## Author’s contribution

Sidra B. Bhuller DO, first author, contributed to the study concept, data collection, data analysis, and writing the paper; Mark Lieser MD and Naveed Ismail reviewed the manuscript; and Bradley Woods MD, senior author and the manuscript reviewer, contributed to the study concept, data analysis, and manuscript.

## Registration of research studies

None.

## Guarantor

Sidra B. Bhuller.

## Provenance and peer review

Not commissioned, externally peer-reviewed.

## Declaration of Competing Interest

None.
